# Microcephaly in South Brazil: Are cases of Congenital Zika Syndrome increasing in recent years?

**DOI:** 10.1590/1678-4685-GMB-2023-0191

**Published:** 2024-01-19

**Authors:** Anna Pires Terra, Ricardo Rohweder, Silvani Herber, Luciana Friedrich, Maria Teresa Vieira Sanseverino, Catia Favreto, Fernanda Santa Maria, Emilly de Jesus Athayde, Laércio Moreira Cardoso-Júnior, Andrea Cristina Pereira Marinho, Allanamara Pereira Marinho, Tailine Zarpelon, Lavínia Schuler-Faccini

**Affiliations:** 1 Universidade Federal do Rio Grande do Sul (UFRGS), Programa de Pós-Graduação em Genética e Biologia Molecular (PPGBM), Porto Alegre, RS, Brazil.; 2 Hospital de Clínicas de Porto Alegre, Serviço de Genética Médica, Sistema de Informação sobre Agentes Teratogênicos (SIAT), Porto Alegre, RS, Brazil.; 3 Hospital de Clínicas de Porto Alegre (HCPA), Serviço de Neonatologia, Porto Alegre, RS, Brazil.; 4 Secretaria de Saúde do Estado do Rio Grande do Sul (SES/RS), Centro Estadual de Vigilância em Saúde (CEVS), Porto Alegre, RS, Brazil.; 5 Universidade Federal do Rio Grande do Sul, Faculdade de Medicina, Porto Alegre, RS, Brazil.; 6 Universidade Federal do Rio Grande do Sul, Faculdade de Odontologia, Porto Alegre, RS, Brazil.; 7 Universidade Federal de Ciências da Saúde de Porto Alegre(UFCSPA), Departamento de Enfermagem, Porto Alegre, RS, Brazil.

**Keywords:** Microcephaly, Zika virus, congenital infection, Congenital Zika Virus Syndrome, surveillance

## Abstract

Northeast Brazil was the first region to detect a significant increase in babies born with microcephaly associated with prenatal zika virus infection in 2015. Rio Grande do Sul (RS) state was less impacted due to the temperate climate preventing the spread of the vector. This study investigated the prevalence and etiology of congenital microcephaly in RS in two different periods. This cross-sectional descriptive study included all live births with congenital microcephaly in RS from 2015 to 2022. Cases were divided into two groups: P1 “outbreak” (2015-16); and P2 “endemic” (2017-22). There were 58 cases of microcephaly (3.8/10,000) in P1 and 148 (1.97/10,000) in P2. Congenital Zika Virus infection was the etiology in 5.2% (n=3) in P1 and 6.7% (n=10) in P2. In conclusion, although the ZIKV outbreak in Brazil has receded, RS remains an area of concern, with a possible slight increase of live births with microcephaly secondary to ZIKV prenatal infection relative to the number of cases due to congenital infections. The broader distribution of the vector *Aedes aegypti* with warmer temperatures in our state might be linked to the increase in recent years. This study can be an alert to other regions of temperate or subtropical climates.

## Introduction

Zika Virus (ZIKV) infection was considered a condition without serious consequences until October 2015, when a sharp increase in newborns (NB) with severe microcephaly was observed in northeastern Brazil (De Oliveira et al., 2016; Schuler-Faccini et al., 2016), followed later by different countries in America Latin America and worldwide (Lowe et al., 2018). Congenital Zika Virus Syndrome (CZS) has been recognized and incorporated into the teratogenic congenital infections known by the acronym STORCH-Z (Rasmussen et al., 2016; Del Campo et al., 2017; França et al. 2018; Proenca-Modena et al., 2018). 

As of 2017, ZIKV infection and microcephaly cases decreased throughout the country, becoming an endemic condition (Brasil, 2023). Therefore, models that help to identify new ZIKV outbreaks or other emerging infectious diseases became critical (Rasmussen et al., 2016; Lowe et al., 2018; Proenca-Modena et al., 2018). Epidemiological surveillance of congenital microcephaly and timely identification of its etiology is essential for detecting new ZIKV outbreaks. Congenital microcephaly in Brazil reached 5.46 cases per 10,000 live births (LB) in 2015. The region with the highest coefficient was the Northeast (13.9/10,000), which corresponds to 28 times the average annual coefficients for this region in the 2000-2014 period (0.5/10,000) (De Oliveira et al., 2016; França et al., 2018; Freitas et al., 2020).

Overall, the number of registered cases of ZIKV in Brazil decreased from 205,578 cases in 2016 to 13,353 in 2017, with population immunity being considered the leading cause of the decline. In 2022, there were 9,256 probable cases up to epidemiological week (SE) 49, corresponding to an incidence rate of 4.3 cases per 100,000 inhabitants in the country (Brasil, 2022).

Rio Grande do Sul (RS) is the southernmost state of Brazil, where a cooler climate prevented an outbreak of ZIKV infection at that time. From December 2015 to December 2017, the prevalence at birth of congenital microcephaly was calculated as 9.6/10,000 live births, with confirmed cases of SCZ representing 5.2% (n=3) (Herber et al., 2019). 

In 2021, there were 265 suspected cases of Zika Virus in RS (45 confirmed by laboratory tests). In 2022 this number was progressively higher, with 482 suspected cases of Zika Virus (57 confirmed) reported (Brasil, 2022). Furthermore, the ongoing transmission of the dengue virus (DENV) in Brazil and the Americas over many decades associated with global climate warming suggests that ZIKV will continue circulating within the human transmission cycle (Van Wyk et al., 2023).

This study aimed to compare data from records of cases of congenital microcephaly already analyzed by Herber et al. (2019) with the post-outbreak period (endemic period) from 2017 to 2022 in RS.

## Subjects and Methods

This is a cross-sectional study analyzing all notifications of liveborn with congenital microcephaly in the state of RS, from December 1, 2015, to December 31, 2016 [Period 1 - Outbreak - data already published by Herber et al., 2019)] and from January 1, 2017, until September 30, 2022 (Period 2 - endemic). Microcephaly was defined as a head circumference below 2 Z-scores, corrected for gender and gestational age, according to the Intergrowth-21st charts (De Oliveira et al., 2017). All newborns were notified in the Public Health Events Registry (RESP) by the health professional who identified the microcephaly (Brasil, 2017) and reviewed by our team. Cases were then classified according to their etiology. Congenital infections were diagnosed considering the following criteria: (1) Congenital Zika Virus Infection - maternal history with clinical findings suggestive of exanthematous infection in pregnancy, a dysmorphological pattern on physical examination, abnormalities on neuroimaging, OR ZIKV polymerase chain reaction (PCR) test positive in the blood; (2) Cytomegalovirus (CMV) - positive PCR in the urine; (3) Toxoplasmosis and Rubella - positive serology for immunoglobulins (IgM) in the blood; (4) Syphilis - positive Venereal Disease Research Laboratory (VDRL) test in blood or cerebrospinal fluid sample or a positive Fluorescent Treponemal Antibody (FTAAbs) test. Serological tests for toxoplasmosis, rubella, and syphilis and PCR for ZIKV of maternal and neonatal samples were performed at the State Central Laboratory (LACEN). VDRL PCR for CMV in urine and neuroimaging tests were performed in the hospital where the child was born or at Hospital de Clínicas de Porto Alegre after referral. Serological, PCR and imaging tests to investigate the etiology of microcephaly cases were funded by the public health system, since they were part of the routine protocol established by the Brazilian Ministry of Health. Some cases were classified as Probable Congenital Zika Virus infection following the definition of the Ministry of Health: when the children have only dysmorphological and imaging abnormalities born from asymptomatic mothers, with inconclusive ZIKV laboratory confirmation, and with classic STORCH tests negative (Brasil, 2017). 

The records were jointly reviewed by the Center for Health Surveillance of the State of Rio Grande do Sul (CEVS) and by the authors at Medical Genetics Service Hospital de Clínicas de Porto Alegre (HCPA) and Genetics Department (Universidade Federal do Rio Grande do Sul). Cases of confirmed microcephaly were referred to the HCPA for clinical evaluation. All were examined by a multidisciplinary team where medical geneticists performed a detailed dysmorphological evaluation and underwent specific genetic tests (karyotyping, array-CGH, genome sequencing and screening for inborn errors of metabolism) following the clinical indication.

The prevalence of microcephaly was calculated through the number of live births in the study period. The spatial distribution of microcephaly cases took the municipality of maternal residence as a reference. Cluster analysis was performed by estimating the spatial correlation of microcephaly cases based on a continuous distance function between municipalities. These analyses were conducted in the R environment using the NCF package version 1.3-2. This study was approved by the Research Ethics Committee of the HCPA, CAEE: 78735817.9.1001.5327.

## Results

In Period 1, when the outbreak of ZIKV was registered in Brazil, 58 cases of microcephaly were reported (3.8/10,000 live births) in RS. In Period 2, 753,143 live births were registered, 148 (2.0/10,000 live births) with congenital microcephaly. Congenital infections remained the main etiological factor identified, accounting for 50.0% of cases in Period 1 and 42.6% in Period 2. Syphilis (P1 22.4% and P2 12.8%), cytomegalovirus (P1 10.3% and P2 13.8%) and toxoplasmosis (P112.1% and P2 5.4%) were the most prevalent (Table 1). ZIKV congenital infection was P1 5.2% and P2 2.0%. Seven additional cases (4.7%) were classified as probable congenital ZIKV infection due to a lack of timely laboratory tests. However, all seven presented the characteristic phenotype and brain imaging. A slight increase in CZS in Period 2 was detected when these cases were considered in the group of congenital infections only (P1=10.3%; P2=15.9%; p=0.70). The 10 CZS cases from Period 2 are described in Table 2. Five of them were born from asymptomatic mothers.

**Table 1 -:** Diagnosis of live newborns reported with microcephaly in Rio Grande do Sul, Brazil: 2015 to 2022.

	2015-2016		2017-2022		
True Microcephaly	N=58	%	N=148	%	P
Congenital Infections	29	50.0	63	42.6	0.418
Syphilis	13	22.4	19	12.8	
Toxoplasmosis	7	12.1	14	9.5	
Cytomegalovirus	6	10.3	20	13.5	
Confirmed Zika Virus	3	5.2	3	2.0	
Probable Zika Virus	-	-	7	4.7	
Chromosomal Disorders	5	8.6	10	6.8	0.766
Trisomy 21	5	8.6	6	4.1	
Trisomy 13	-	-	1	0.7	
Trisomy 18	-	-	1	0.7	
Ring chromosome 13	-	-	1	0.7	
Cri Du Chat Syndrome	-	-	1	0.7	
Monogenic disorders	4	6.9	13	8.8	0.784
Fetal Alcohol Syndrome	-	-	1	0.7	
Unknown diagnosis/under investigation	11	19.0	24	16.2	0.790
Isolated CNS abnormalities^a^	9	15.5	37	25.0	0.199
False Positives^b^	90/148	60.8	180/336	53.6	0.168
Lost / No sufficient data	-	-	8/336	2.4	

^a^ CNS (Central Nervous System).

^b^ False positives: cases initially reported but excluded for the following reasons: Head Circumference above -2Z scores 48hs after birth and baby without neurological problems, OR adjusting for sex and gestational age (*Intergrowth 21* ^*st*^ ).

**Table 2 -:** Characteristics of cases with confirmed or suspected ZIKV syndrome in period 2.

Case	Sex	Year of Birth	GA^a^	HC^b^	Z score^c^	Trimester^d^	CZS Phenotype	NPMD^e^	MRI/CT^f^ compatible
1	F	2017	37	28	-3.5	1º	Yes	Delay	Yes
2	F	2017	37	30	-2.2	2º	Yes	Delay	Yes
3	M	2017	40	29	-3.9	NI	Yes	Delay	Yes
4	F	2017	38	29	-3.2	NI	Yes	Delay	Yes
5	M	2017	40	30	-3.6	3º	Yes	Delay	Yes
6	M	2017	38	31	-2.0	3º	Yes	Delay	Yes
7	M	2018	35	29.5	-2.0	NI	Yes	Delay	Yes
8	F	2018	36	27	-3.7	NI	Yes	Delay	Yes
9	F	2018	37	32	-0.4	3º	Yes	Delay	Yes
10	F	2018	35	30	-1.6	NI	Yes	Delay	Yes

^a^ Gestational age (weeks).

^b^ Head circumference at birth (cm).

^c^ Z-score according to InterGrowth-21^st^

^d^ Gestational trimester of symptoms; NI (not identified) means asymptomatic women.

^e^ Neuropsychomotor development.

^f^ Magnetic resonance imaging of the skull and tomography of the skull.

Genetic conditions associated with microcephaly were identified in 15.6% of all patients in P2, both chromosomal (6.8%) and monogenic (8.8%). Although genetic tests were available, 16.2% of patients remained without a clear etiological classification; some are still waiting for the genome analysis. Isolated multifactorial brain anomalies were also common (25.5%), and included holoprosencephaly, anencephaly, occipital encephalocele and Dandy-Walker anomaly.

**Figure 1 -:**
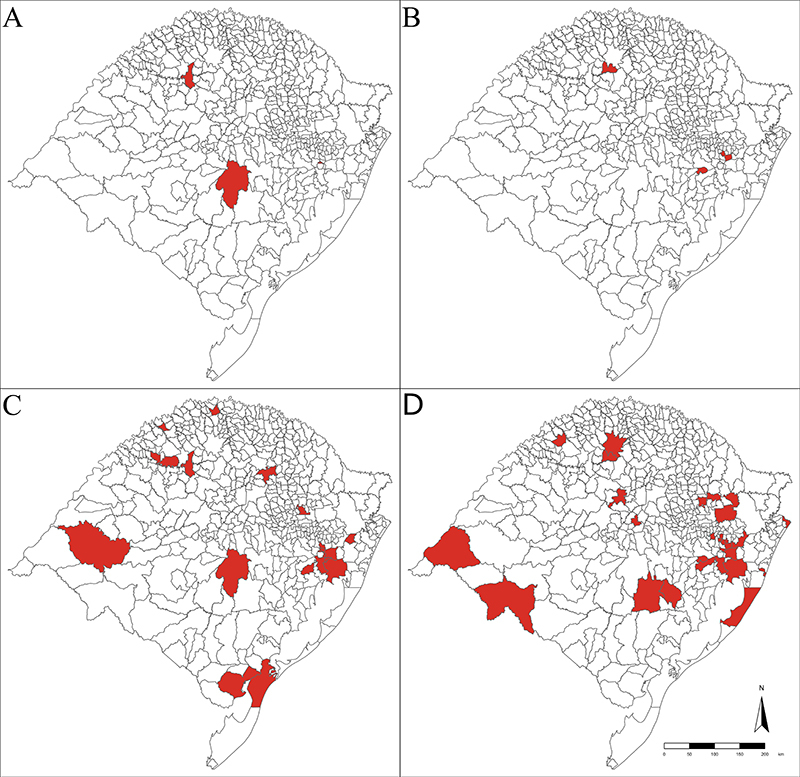
Spatial distribution of microcephaly cases due to congenital infection in Rio Grande do Sul, based on the maternal municipality of residence, from December 1, 2015, to December 31, 2016 (Period 1) and from January 1, 2017 to September 30, 2022 (Period 2). A - Municipalities with cases of microcephaly due to congenital Zika virus infection during Period 1; B - Municipalities with cases of microcephaly due to congenital Zika virus infection during Period 2; C - Municipalities with cases of microcephaly due to congenital infections (syphilis, toxoplasmosis, cytomegalovirus, and Zika virus) during period 1; D - Municipalities with cases of microcephaly due to congenital infections (syphilis, toxoplasmosis, cytomegalovirus, and Zika virus) during Period 2.

**Figure 2 -:**
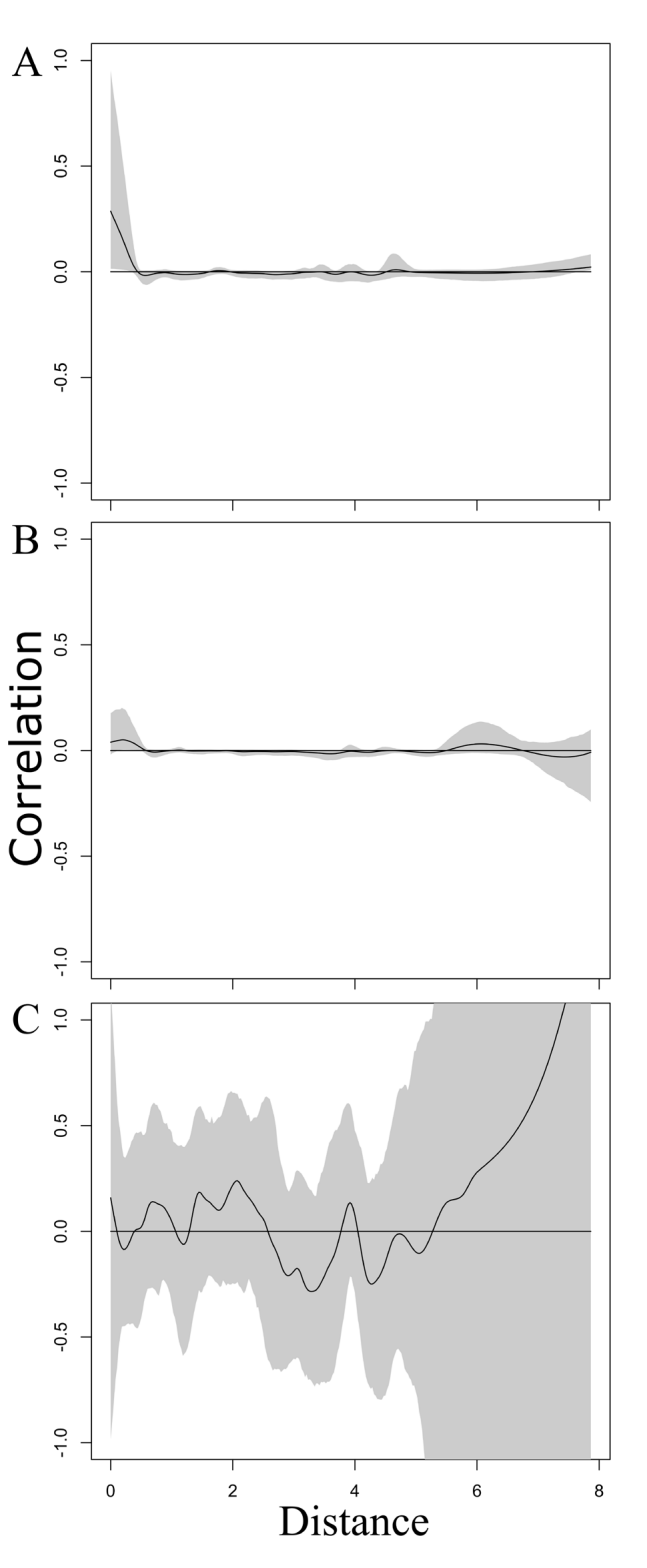
Spatial and temporal correlation of microcephaly cases due to congenital infection, based on the maternal municipality of residence, from December 1, 2015, to December 31, 2016 (Period 1) and from January 1, 2017, to September 30, 2022 (Period 2). A - Correlation of the spatial distribution of microcephaly cases due to congenital infection during Period 1; B - Correlation of the spatial distribution of microcephaly cases due to congenital infection during Period 2; C - Correlation of the spatial and temporal distribution of microcephaly cases due to congenital infection between Period 1 and Period 2. The grey area represents the 95% confidence interval, and the solid black line represents the mean of the estimated correlation coefficient. The distance is given in degrees, where one degree corresponds to approximately 90 kilometres in Rio Grande do Sul.

The cases of microcephaly due to congenital Zika virus infection came from different municipalities (Figure 1A, B), as well as the cases whose etiology was attributed to other congenital infections (Figure 1C, D). There is one weak spatial correlation between very close municipalities with cases of microcephaly due to congenital infection in the two evaluated periods (Figure 2A, B). Still, when comparing the two periods, there is no evidence of correlation (Figure 2C ).

## Discussion

Between October 2015 and May 2017, 26 countries in the Americas reported confirmed cases of congenital ZIKV syndrome. During this period, 3,374 cases (82%) occurred in Brazil (Albuquerque et al., 2018). In Northeast Brazil, a peak prevalence of 49/10,000 live births was recorded (De Oliveira et al., 2017). In contrast, the prevalence of microcephaly in newborns in RS was only 3.8/10,000 in 2015-16 but still well above the registered 0.5/10,000 in Brazil before the outbreak of microcephaly caused by ZIKV (De Oliveira *et al.*, 2017; Herber et al., 2019).

Reporting of microcephaly cases has shown a decline since the end of the public health emergency period in 2017. Besides populational acquired immunity, the decrease in total cases of microcephaly can be attributed to lower notification by health professionals of patients with a phenotype not suggestive of congenital infection. Therefore, surveillance remains active to identify new cases and deaths in the country (Brasil, 2022). Discrepancies in the prevalence estimates might also be attributable to different criteria of head circumference thresholds to define congenital microcephaly (Victora et al., 2016). Castro et al. (2018) analyzed the temporal distribution of CZS in Brazil, showing a decrease in new cases from May 2016. Out of 2,751 confirmed cases of CZS since 2015, only 76 (2.8%) were born in 2017. 

The present study compared two periods of notification of microcephaly in Rio Grande do Sul state. In 2015-16, 50.0% of cases of microcephaly were attributed to congenital infection, but only 10.3% among them were secondary to ZIKV. From 2017 to 2022, 42.6% of the cases were related to infection, but 15.9% were secondary to confirmed cases or probable ZIKV.

In 2016, 42.4% of 497 municipalities in RS were declared to be infested with *Aedes aegypti.* In 2022 this percentage rose to 91.0%. Dengue fever shares the same vector as Zika and is another good proxy for arboviral infection distribution. In 2016, in RS, 2.437 cases of dengue were reported in RS; in 2022, there were 66.779 reported cases (Rio Grande do Sul, 2022). Zika fever is also a mandatory report in Brazil. However, it is usually underreported since their symptoms are generally mild or asymptomatic in up to 80% of the cases (Haby et al., 2018). Besides that, laboratory confirmation for ZIKV still represents a challenge: RT-PCR in the blood is the gold standard but is only performed in symptomatic individuals, and its sensitivity is limited due to the short viremia period. Serological tests present a broad range of cross-reactivity with other flaviviruses, especially in dengue-pre-exposed individuals. PRNT (plaque reduction neutralization test) is a serological test that is more sensitive, but it is costly, time-demanding, and not available at public health facilities (de Vasconcelos *et al.*, 2018). RS registered only 85 cases of Zika fever in 2016 and 57 in 2022 (Rio Grande do Sul, 2022).

Of the 10 cases described here, five were born from asymptomatic mothers. In seven, we applied the clinical-epidemiological criteria for diagnosing congenital zika syndrome without laboratory confirmation. This leads to contradictory data, where the number of maternal infections in pregnancy is possibly lower than the cases diagnosed in newborns. In this context, a detailed and careful dysmorphological and clinical examination, with imaging exams, has a decisive role in detecting SCZ cases (Schuler-Faccini et al., 2016; Graham et al., 2017; Reynolds et al., 2017).

We did not observe an evident pattern of spatial correlation among the cases of microcephaly secondary to ZIKV prenatal infection. Compared to what was observed in other states during the outbreaks of 2015 and 2016 (De Oliveira et al., 2017), a smaller number of microcephaly cases caused by Zika virus infection were observed in RS. These cases originated from different state regions and do not form a cluster. Despite that, the high prevalence of infestation by the mosquito vector *Aedes aegypti*, along with the increasing number of arbovirus infections each year (Tumioto et al., 2014; Gregianini et al., 2017), positions RS as a conducive environment for the spread of ZIKV infections and susceptible to outbreaks. Statistical modelling studies have been instrumental in identifying sensitive regions, a crucial step in planning resource allocation, devising preventive healthcare measures, and directing research endeavors (Kraemer et al., 2019). Rio Grande do Sul is one of these regions, with a population that remains potentially susceptible to this infection and its consequences, particularly concerning pregnant women.

Our study has some limitations: only live births and only those diagnosed with microcephaly at birth or immediately after were included. An absence of signs and symptoms at birth in exposed babies does not rule out ZIKV congenital infection with late-onset manifestations, especially visual (Freitas et al., 2020; Ventura et al., 2021; Merle et al., 2022; Rosado et al., 2023).

The social and economic impacts of CZS are severe and lasting. It is understood, therefore, that a continuous international response and intensified and interdisciplinary research is needed to improve the ability to anticipate, control and mitigate the risk of ZIKV and other re-emerging and emerging arboviruses that constitute threats to public health (Costa and Ko, 2018). 

In conclusion, the ZIKV outbreak in Brazil has receded, but RS remains an area risk with a possible increase in cases of microcephaly in the post-outbreak period. With warmer temperatures and the vector’s spread as observed in RS from 2016 to the present day, surveillance for cases of ZIKV infection and microcephaly should remain active. 
